# *N*,*N*-Dimethyl-*N*′-[3-(trifluoro­methyl)­phenyl]­urea

**DOI:** 10.1107/S1600536808016656

**Published:** 2008-06-07

**Authors:** Da-sheng Yu, Fang-shi Li, Wei Yao, Yin-hong Liu, Chui Lu

**Affiliations:** a Department of Applied Chemistry, College of Science, Nanjing University of Technolgy, Xinmofan Road No.5 Nanjing, Nanjing 210009, People’s Republic of China

## Abstract

The title compound, C_10_H_11_F_3_N_2_O, is an important urea-based herbicide. In the crystal structure, the mol­ecular packing is stabilized by two intra­molecular C—H⋯O hydrogen bonds and one inter­molecular N—H⋯O hydrogen bond, generating a *C*(4) graph-set motif running parallel to the [001] direction. The F atoms are disordered over two sites, with occupancies of 0.176 (9) and 0.824 (9).

## Related literature

For related literature, see: Bernstein *et al.* (1995 ▶); Xu *et al.* (2005 ▶); Zhao & Wilkins (2003 ▶); Li *et al.* (2007 ▶).
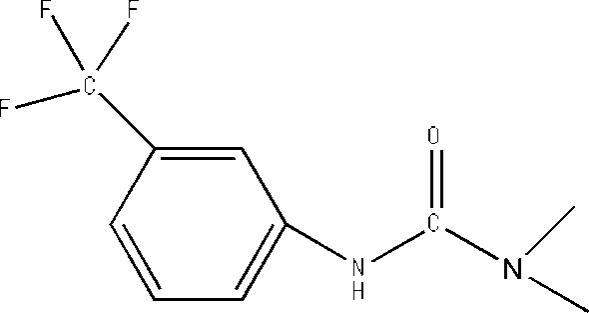


## Experimental

### 

#### Crystal data

C_10_H_11_F_3_N_2_O
*M* *_r_* = 232.20
Monoclinic, 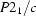

*a* = 11.005 (2) Å
*b* = 9.991 (2) Å
*c* = 10.012 (2) Å
β = 96.89 (3)°
*V* = 1092.9 (4) Å^3^
*Z* = 4
Mo *K*α radiation
μ = 0.13 mm^−1^
*T* = 298 (2) K
0.30 × 0.20 × 0.10 mm

#### Data collection

Enraf–Nonius CAD4 diffractometer
Absorption correction: multi-scan (North *et al.*, 1968 ▶) *T*_min_ = 0.963, *T*_max_ = 0.987
2076 measured reflections
1953 independent reflections
1335 reflections with *I* > 2σi(*I*)
*R*_int_ = 0.020
3 standard reflections every 200 reflections intensity decay: none

#### Refinement

*R*[*F* ^2^ > 2σ(*F* ^2^)] = 0.053
*wR*(*F* ^2^) = 0.137
*S* = 1.00
1953 reflections
169 parameters
36 restraints
H-atom parameters constrained
Δρ_max_ = 0.18 e Å^−3^
Δρ_min_ = −0.15 e Å^−3^

### 

Data collection: *CAD-4 Software* (Enraf–Nonius, 1989 ▶); cell refinement: *CAD-4 Software*; data reduction: *XCAD4* (Harms & Wocadlo, 1995 ▶); program(s) used to solve structure: *SHELXS97* (Sheldrick, 2008 ▶); program(s) used to refine structure: *SHELXL97* (Sheldrick, 2008 ▶); molecular graphics: *SHELXTL* (Sheldrick, 2008 ▶); software used to prepare material for publication: *SHELXTL*.

## Supplementary Material

Crystal structure: contains datablocks I. DOI: 10.1107/S1600536808016656/bx2148sup1.cif

Structure factors: contains datablocks I. DOI: 10.1107/S1600536808016656/bx2148Isup2.hkl

Additional supplementary materials:  crystallographic information; 3D view; checkCIF report

## Figures and Tables

**Table 1:** Hydrogen-bond geometry (Å, °)

*D*—H⋯*A*	*D*—H	H⋯*A*	*D*⋯*A*	*D*—H⋯*A*
N1—H1*A*⋯O^i^	0.86	2.08	2.880 (3)	155
C3—H3*A*⋯O	0.93	2.48	2.884 (3)	106
C9—H9*A*⋯O	0.96	2.28	2.721 (4)	107

Symmetry code: (i) 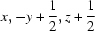.

**Fig. 1.**
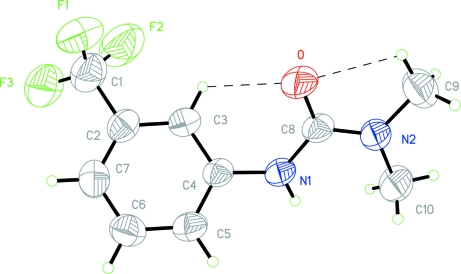
The molecular structure of (I), with the atom-numbering scheme. Displacement ellipsoids are drawn at the 50% probability level.

**Fig. 2.**
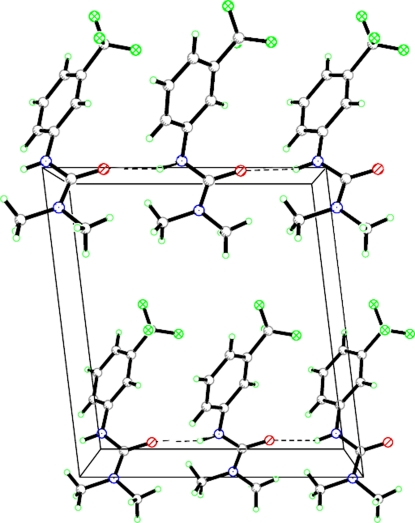
The molecule packing diagram.

## supplementary crystallographic information

### Comment

The title compound, (I), is a pre- and postemergence herbicide used widely as water dispersible and suspension concentrate formulations for the control of grass and broadleaf weeds in cotton and sugarcane (Zhao & Wilkins, 2003). As part of our studies in this area (Li *et al.*, 2007), we report herein the crystal structure of the title compound, (I), Fig 1. In the crystal structure the molecular packing is stabilized by two intramolecular C—H···O as well as one intermolecular N—H···O hydrogen bond generating a graph-set motif C(4) running parallel to [001] direction (Bernstein *et al.*, 1995), Table 1.

### Experimental

The title compound, (I), was prepared according to the literature method (Xu *et al.*, 2005). The crystals suitable for X-ray analysis were obtained by dissolving (I) (0.1 g, in acetonitrile (25 ml) and evaporating the solvent slowly at room temperature for about 7 d.

### Refinement

H atoms were positioned geometrically, C—H = 0.86, 0.93 and 0.96 Å for amido, aromatic and methyl H, and constrained to ride on their parent atoms, with *U*_iso_(H) = *xU*_eq_(C,*N*), where *x* = 1.5 for methyl H, and *x* = 1.2 for all other H atoms.

Trifloromethyl group was disordered over two sites, occupancies were refined and converged to 0.176 (9) and 0.824 (9), respectively.

### Crystal data

**Table:**

C_10_H_11_F_3_N_2_O	*F*_000_ = 480
*M**_r_* = 232.20	*D*_x_ = 1.411 Mg m^−^^3^
Monoclinic, *P*2_1_/*c*	Mo *K*α radiation λ = 0.71073 Å
Hall symbol: -P 2ybc	Cell parameters from 25 reflections
*a* = 11.005 (2) Å	θ = 10–13º
*b* = 9.991 (2) Å	µ = 0.13 mm^−^^1^
*c* = 10.012 (2) Å	*T* = 298 (2) K
β = 96.89 (3)º	Needle, colourless
*V* = 1092.9 (4) Å^3^	0.30 × 0.20 × 0.10 mm
*Z* = 4	

### Data collection

**Table:**

Enraf–Nonius CAD4 diffractometer	*R*_int_ = 0.020
Radiation source: fine-focus sealed tube	θ_max_ = 25.2º
Monochromator: graphite	θ_min_ = 1.9º
*T* = 298(2) K	*h* = −13→13
ω/2θ scans	*k* = −11→0
Absorption correction: multi-scan(North *et al.*, 1968)	*l* = 0→11
*T*_min_ = 0.963, *T*_max_ = 0.987	3 standard reflections
2076 measured reflections	every 200 reflections
1953 independent reflections	intensity decay: none
1335 reflections with *I* > 2σi(*I*)	

### Refinement

**Table:**

Refinement on *F*^2^	Secondary atom site location: difference Fourier map
Least-squares matrix: full	Hydrogen site location: inferred from neighbouring sites
*R*[*F*^2^ > 2σ(*F*^2^)] = 0.053	H-atom parameters constrained
*wR*(*F*^2^) = 0.137	*w* = 1/[σ^2^(*F*_o_^2^) + (0.050*P*)^2^ + 0.630*P*] where *P* = (*F*_o_^2^ + 2*F*_c_^2^)/3
*S* = 1.00	(Δ/σ)_max_ < 0.001
1953 reflections	Δρ_max_ = 0.19 e Å^−^^3^
169 parameters	Δρ_min_ = −0.15 e Å^−^^3^
36 restraints	Extinction correction: SHELXL97 (Sheldrick, 2008), Fc^*^=kFc[1+0.001xFc^2^λ^3^/sin(2θ)]^-1/4^
Primary atom site location: structure-invariant direct methods	Extinction coefficient: 0.162 (9)

### Special details

**Table:**

Geometry. All e.s.d.'s (except the e.s.d. in the dihedral angle between two l.s. planes) are estimated using the full covariance matrix. The cell e.s.d.'s are taken into account individually in the estimation of e.s.d.'s in distances, angles and torsion angles; correlations between e.s.d.'s in cell parameters are only used when they are defined by crystal symmetry. An approximate (isotropic) treatment of cell e.s.d.'s is used for estimating e.s.d.'s involving l.s. planes.
Refinement. Refinement of *F*^2^ against ALL reflections. The weighted *R*-factor *wR* and goodness of fit *S* are based on *F*^2^, conventional *R*-factors *R* are based on *F*, with *F* set to zero for negative *F*^2^. The threshold expression of *F*^2^ > σ(*F*^2^) is used only for calculating *R*-factors(gt) *etc*. and is not relevant to the choice of reflections for refinement. *R*-factors based on *F*^2^ are statistically about twice as large as those based on *F*, and *R*- factors based on ALL data will be even larger.

### Fractional atomic coordinates and isotropic or equivalent isotropic displacement parameters (Å^2^)

**Table:**

	*x*	*y*	*z*	*U*_iso_*/*U*_eq_	Occ. (<1)
C1	−0.4416 (3)	0.3310 (5)	0.7481 (4)	0.083	
F1	−0.4245 (19)	0.355 (3)	0.6336 (15)	0.097 (6)	0.176 (9)
F2	−0.444 (2)	0.1924 (18)	0.754 (3)	0.127 (8)	0.176 (9)
F3	−0.5413 (13)	0.368 (3)	0.773 (2)	0.102 (6)	0.176 (9)
F1'	−0.4988 (5)	0.4328 (5)	0.6794 (6)	0.149 (2)	0.824 (9)
F2'	−0.4081 (3)	0.2491 (7)	0.6573 (5)	0.126 (2)	0.824 (9)
F3'	−0.5304 (4)	0.2692 (7)	0.8022 (4)	0.1244 (19)	0.824 (9)
O	−0.00769 (19)	0.2044 (2)	0.79738 (17)	0.0702 (7)	
N1	−0.0375 (2)	0.2618 (2)	1.0096 (2)	0.0532 (7)	
H1A	−0.0106	0.2548	1.0935	0.064*	
N2	0.1237 (2)	0.1254 (3)	0.9701 (2)	0.0569 (7)	
C2	−0.3406 (3)	0.3766 (3)	0.8510 (3)	0.0553 (8)	
C3	−0.2362 (2)	0.2993 (3)	0.8761 (3)	0.0508 (7)	
H3A	−0.2280	0.2211	0.8277	0.061*	
C4	−0.1437 (2)	0.3397 (3)	0.9745 (2)	0.0460 (7)	
C5	−0.1575 (3)	0.4563 (3)	1.0448 (3)	0.0572 (8)	
H5A	−0.0954	0.4840	1.1102	0.069*	
C6	−0.2618 (3)	0.5322 (3)	1.0194 (3)	0.0649 (9)	
H6A	−0.2703	0.6103	1.0680	0.078*	
C7	−0.3543 (3)	0.4927 (3)	0.9216 (3)	0.0627 (9)	
H7A	−0.4249	0.5439	0.9037	0.075*	
C8	0.0253 (2)	0.1970 (3)	0.9195 (2)	0.0492 (7)	
C9	0.1950 (3)	0.0560 (4)	0.8790 (3)	0.0814 (11)	
H9A	0.1592	0.0706	0.7879	0.122*	
H9B	0.1957	−0.0381	0.8983	0.122*	
H9C	0.2773	0.0896	0.8903	0.122*	
C10	0.1652 (3)	0.1137 (4)	1.1124 (3)	0.0765 (11)	
H10A	0.0972	0.0913	1.1599	0.115*	
H10B	0.1997	0.1974	1.1454	0.115*	
H10C	0.2262	0.0448	1.1264	0.115*	

### Atomic displacement parameters (Å^2^)

**Table:**

	*U*^11^	*U*^22^	*U*^33^	*U*^12^	*U*^13^	*U*^23^
C1	0.059	0.100	0.086	−0.002	−0.002	−0.005
F1	0.102 (10)	0.117 (11)	0.066 (7)	−0.013 (8)	−0.012 (6)	−0.014 (7)
F2	0.118 (11)	0.114 (10)	0.134 (12)	−0.017 (8)	−0.041 (8)	−0.013 (8)
F3	0.052 (7)	0.134 (11)	0.115 (10)	−0.004 (8)	−0.004 (6)	−0.016 (9)
F1'	0.135 (4)	0.122 (3)	0.162 (4)	0.004 (3)	−0.093 (3)	0.022 (3)
F2'	0.073 (2)	0.192 (5)	0.105 (3)	0.005 (3)	−0.0191 (18)	−0.077 (4)
F3'	0.081 (2)	0.153 (4)	0.140 (3)	−0.058 (3)	0.014 (2)	−0.016 (3)
O	0.0692 (13)	0.1123 (19)	0.0284 (10)	0.0120 (12)	0.0031 (9)	−0.0004 (10)
N1	0.0590 (14)	0.0718 (16)	0.0269 (10)	0.0113 (13)	−0.0028 (10)	−0.0003 (11)
N2	0.0562 (14)	0.0701 (17)	0.0433 (13)	0.0112 (13)	0.0017 (11)	−0.0004 (12)
C2	0.0476 (16)	0.067 (2)	0.0508 (16)	−0.0061 (15)	0.0040 (13)	0.0053 (15)
C3	0.0542 (17)	0.0543 (17)	0.0430 (15)	−0.0044 (14)	0.0027 (12)	−0.0013 (13)
C4	0.0501 (15)	0.0563 (17)	0.0312 (12)	0.0004 (13)	0.0040 (11)	0.0034 (12)
C5	0.0628 (18)	0.066 (2)	0.0417 (15)	−0.0010 (16)	0.0002 (13)	−0.0058 (14)
C6	0.074 (2)	0.063 (2)	0.0579 (18)	0.0033 (17)	0.0088 (16)	−0.0090 (15)
C7	0.0570 (18)	0.068 (2)	0.0637 (19)	0.0106 (16)	0.0093 (15)	0.0088 (17)
C8	0.0505 (16)	0.0645 (18)	0.0323 (14)	−0.0022 (14)	0.0039 (11)	0.0020 (12)
C9	0.078 (2)	0.098 (3)	0.071 (2)	0.022 (2)	0.0198 (18)	−0.004 (2)
C10	0.079 (2)	0.095 (3)	0.0517 (18)	0.023 (2)	−0.0079 (16)	0.0085 (18)

### Geometric parameters (Å, °)

**Table:**

C1—F1	1.208 (16)	C2—C3	1.382 (4)
C1—F3	1.214 (15)	C3—C4	1.389 (4)
C1—F2'	1.308 (5)	C3—H3A	0.9300
C1—F3'	1.325 (6)	C4—C5	1.380 (4)
C1—F1'	1.341 (6)	C5—C6	1.374 (4)
C1—F2	1.386 (18)	C5—H5A	0.9300
C1—C2	1.494 (5)	C6—C7	1.383 (4)
O—C8	1.235 (3)	C6—H6A	0.9300
N1—C8	1.364 (3)	C7—H7A	0.9300
N1—C4	1.412 (3)	C9—H9A	0.9600
N1—H1A	0.8600	C9—H9B	0.9600
N2—C8	1.345 (3)	C9—H9C	0.9600
N2—C10	1.448 (4)	C10—H10A	0.9600
N2—C9	1.449 (4)	C10—H10B	0.9600
C2—C7	1.376 (4)	C10—H10C	0.9600
			
F1—C1—F3	112.7 (14)	C2—C3—C4	119.3 (3)
F1—C1—F2'	51.4 (11)	C2—C3—H3A	120.3
F3—C1—F2'	132.5 (9)	C4—C3—H3A	120.3
F1—C1—F3'	133.4 (8)	C5—C4—C3	119.4 (3)
F3—C1—F3'	47.9 (13)	C5—C4—N1	118.5 (2)
F2'—C1—F3'	106.1 (5)	C3—C4—N1	122.1 (2)
F1—C1—F1'	58.6 (12)	C6—C5—C4	120.8 (3)
F3—C1—F1'	59.6 (12)	C6—C5—H5A	119.6
F2'—C1—F1'	105.8 (5)	C4—C5—H5A	119.6
F3'—C1—F1'	103.9 (4)	C5—C6—C7	120.1 (3)
F1—C1—F2	104.2 (15)	C5—C6—H6A	120.0
F3—C1—F2	106.2 (16)	C7—C6—H6A	120.0
F2'—C1—F2	54.1 (12)	C2—C7—C6	119.2 (3)
F3'—C1—F2	60.0 (12)	C2—C7—H7A	120.4
F1'—C1—F2	140.5 (8)	C6—C7—H7A	120.4
F1—C1—C2	113.9 (8)	O—C8—N2	122.2 (3)
F3—C1—C2	112.2 (9)	O—C8—N1	120.9 (3)
F2'—C1—C2	114.9 (3)	N2—C8—N1	116.9 (2)
F3'—C1—C2	112.6 (4)	N2—C9—H9A	109.5
F1'—C1—C2	112.7 (4)	N2—C9—H9B	109.5
F2—C1—C2	106.8 (8)	H9A—C9—H9B	109.5
C8—N1—C4	124.5 (2)	N2—C9—H9C	109.5
C8—N1—H1A	117.7	H9A—C9—H9C	109.5
C4—N1—H1A	117.7	H9B—C9—H9C	109.5
C8—N2—C10	123.9 (2)	N2—C10—H10A	109.5
C8—N2—C9	119.3 (2)	N2—C10—H10B	109.5
C10—N2—C9	116.8 (3)	H10A—C10—H10B	109.5
C7—C2—C3	121.2 (3)	N2—C10—H10C	109.5
C7—C2—C1	119.6 (3)	H10A—C10—H10C	109.5
C3—C2—C1	119.3 (3)	H10B—C10—H10C	109.5

### Hydrogen-bond geometry (Å, °)

**Table:**

*D*—H···*A*	*D*—H	H···*A*	*D*···*A*	*D*—H···*A*
N1—H1A···O^i^	0.86	2.08	2.880 (3)	155
C3—H3A···O	0.93	2.48	2.884 (3)	106
C9—H9A···O	0.96	2.28	2.721 (4)	107

Symmetry codes: (i) *x*, −*y*+1/2, *z*+1/2.
